# Evaluating network adequacy of oral health services for children on Medicaid in Arizona

**DOI:** 10.3934/publichealth.2022005

**Published:** 2021-11-16

**Authors:** Swapna Reddy, Matthew Speer, Mary Saxon, Madison Ziegler, Zaida Dedolph, Siman Qaasim

**Affiliations:** 1 College of Health Solutions, Arizona State University, Phoenix, AZ, USA; 2 Children's Action Alliance, Phoenix, AZ, USA

**Keywords:** health disparities, access to care, government programs and policies, oral health, network adequacy

## Abstract

**Purpose:**

Inadequate networks can prevent patients from being able to see the providers that they trust and depend upon, especially for children insured through Medicaid. To improve our understanding of poor oral health care outcomes, we conducted a test of network adequacy among Medicaid pediatric dental providers in Arizona through a “secret shopper” phone survey.

**Methods:**

This study tested multiple components of children's access to oral health care, including reliability of provider directory information, appointment availability at the practice level for children covered under Medicaid versus commercial insurance, and compliance with regulatory standards. We contacted individual providers, following a standardized script to schedule a routine appointment on behalf of a 5-year-old patient enrolled in either a Medicaid or commercial plan. We documented the time until the next available appointment, if the practice was reached, and if the practice accepted the specified insurance plan.

**Results:**

We identified, catalogued, and attempted to call a total of 185 unique practices across Arizona. In four counties, we were unable to identify a single pediatric oral health provider through health plan directories. We observed minimal differences in appointment wait times between callers with commercial insurance and those insured through Medicaid.

**Conclusions:**

Our findings underscore the need to improve the accessibility of pediatric health services, especially in rural regions. Facilitating access to routine and recommended oral health screenings for children enrolled in Medicaid is imperative to appropriate stewardship and fulfilling our commitment to provide this vital public health resource.

## 1. Introduction

In keeping with the original intent of the Medicaid program in the United States to improve access to care for vulnerable populations, the Centers for Medicare & Medicaid Services (CMS) launched the National Oral Health Initiative in 2010 to support states in their efforts to improve utilization of preventive oral health services among Medicaid-enrolled children [1]. Yet while all states must provide dental benefits to children covered by the Medicaid program [2], pervasive oral health disparities and low utilization of services persist—especially among racial and ethnic minorities and rural communities. In order to move the needle forward in meeting the promise of the Medicaid program, it is critical that we increase access to and utilization of key preventive measures such as oral health services for our most vulnerable children. A key step in doing so requires that state administrators and policymakers maintain adequate and accessible networks, otherwise known as groups of dental care providers, that are contracted to provide services to enrollees.

Medicaid plays an especially critical role for children's health in large states with diverse populations. More than 2.1 million Arizonans were enrolled in the state's Medicaid program, the Arizona Health Care Cost Containment Agency (AHCCCS), as of January 2021—up from roughly 1.9 million the previous year [3]. This represents more than 27% of the state's entire population, much higher than the national average of 22%. Of those on Medicaid in Arizona, about 2 in 5 are children, 1 in 3 identify as Hispanic or Latino, and more than 8% are Native American, all contributing to one of the most diverse and fastest-growing state populations in the nation [3]. Only 2 of Arizona's 15 counties are generally considered urban (Maricopa County and Pima County) and the remaining 13 are mostly rural [4]. Arizona also has more tribal land than any other state in the country, comprising more than 27% of the state by area [5]. This confluence of unique demographic and geographic factors underscores both the needs and challenges involved in properly maintaining health networks for patients and their families.

### 1.1. Oral health: a critical component of a child's health

Though all too often overlooked when measuring the overall health of children, oral health is a foundational pillar of every child's overall health and well-being. Research has shown that poor oral health outcomes in the form of tooth decay, including cavities and dental caries, can have a profound detrimental impact on a child's development, quality of life, and school performance lifespan [6]–[7]. Low-income children are at higher risk for developing poor oral health outcomes. In children ages 2–5 years, rates as high as 42% of low-income children had dental caries in their primary teeth, compared to 18% of children from higher income families [8]. School-age children from lower socioeconomic status families are twice as likely to develop cavities due to poor oral health, as compared with their counterparts from higher-income families [9]. Early prevention efforts and regular oral health care are critical to prevent or eradicate tooth decay in children.

Despite ongoing efforts to address these oral health disparities among children in Arizona, they persist. A 2015 statewide survey of kindergarten and third grade children attending Arizona's public schools showed that 52% of the state's kindergarteners had a history of tooth decay, compared to the national average of 36% [10]. The prevalence of third grade children with at least one dental sealant in Arizona was also substantially higher than the general U.S. population—44% versus 32%. These disparities were particularly pronounced in Native American and Hispanic children, especially those attending lower income schools.

### 1.2. Network adequacy: a key component to access

Network Adequacy refers to a health plan's ability to deliver covered medical care by providing reasonable access to all covered services for enrollees, through a sufficient supply of contracted providers. According to federal law [11], Medicaid managed care plans must provide timely access to all covered services through both in-network and out-of-network providers. The law stipulates that timely and equitable access to these services must be made available to beneficiaries and include a broad range of preventive services. Though federal law establishes a broad framework of network adequacy, it is up to states to develop more detailed standards for access and availability for state-contracted Managed Care Organizations (MCOs). These standards include time and distance (e.g., from residence to practice), provider-to-member ratios, and appointment scheduling and wait times. These standards are critical for determining the availability and accessibility of preventive services to Medicaid participants.

However, very few states have clear and transparent standards or metrics in place to evaluate and report network adequacy [12]. This flexibility has led to a patchwork of enforcement standards across the country, thus diluting the effectiveness of these laws. The federal government requires documentation of these standards to be easily accessible, though policies adopted by state Medicaid programs are not consistently operationalized.

Previous research has also demonstrated large disparities in scheduling a variety of health care appointments for Medicaid patients compared with privately insured patients [13]. Especially for children on Medicaid, inadequate networks can prevent patients from being able to see the providers that they know, trust, and depend upon throughout their lives. Continuity of care is vital to ensuring access to key preventive health services and fulfilling our duty to provide timely, high-quality care to children who receive health care through Medicaid.

This case study attempts to evaluate the adequacy of pediatric oral health networks for children on Medicaid in Arizona. Methods and findings may be replicated or scaled in similar communities nationally to inform efforts to reduce a variety of health disparities, especially among particularly vulnerable populations.

## 2. Methods

This study was designed to test the network adequacy of Arizona's pediatric dental provider network through a “secret shopper” phone survey conducted through calls to Medicaid-contracted pediatric practices. The phone survey tested various components of children's access to oral health care through a standardized script of questions, including reliability of provider directory information, appointment availability at the practice level for children enrolled in Medicaid and those with commercial insurance, and compliance with regulatory standards. Existing literature supports this research method as an ethical means of testing the compliance of public programs with government-enforced regulatory standards [14]. AHCCCS, Arizona's Medicaid program, has contracts with seven MCOs across three Geographic Service Areas (GSAs) in Arizona (Central, North, and South). For both commercially-insured and Medicaid-enrolled patients, the health plan with the most robust provider networks within each of the three AHCCCS-designated GSAs was chosen. Following the selection of MCO plans, researchers developed a comprehensive directory of all explicitly designated “pediatric” dental practices within each GSA as identified from the selected plan's website directory which included the practice location, hours of operation, contracted providers, and details regarding the type of insurance accepted. Providers were each matched with one managed care and one commercial insurance plan to ensure consistency across the directory. In rural counties where provider networks are limited, researchers searched multiple MCO plans to identify pediatric dental practices if none within the originally chosen MCO plan were available.

Between January and March 2020, researchers contacted each practice on the directory, following a standardized script and procedures (see Appendix). The script was developed and approved as part of the research protocol filed through the ASU Institutional Review Board. Each provider received one call on behalf of a 5-year-old patient enrolled in a Medicaid managed care plan and one call on behalf of a 5-year-old patient enrolled in a commercial insurance plan. For each call, research assistants assessed and documented in the directory whether the practice could be reached, whether the practice took the predetermined insurance plan, and whether the practice was accepting new patients. If so, researchers inquired about the next available appointment date. Calls to the same practice on behalf of a commercially-insured and Medicaid-insured patient were conducted within 48 hours of each other for consistency and to reduce sampling error. Researchers documented the number of days until the next available appointment, whether the practice was reachable by phone, and whether the practice did in fact accept the specified insurance plan. If the researcher was not able to reach the practice upon first call, they did not leave a voicemail message but did document that the call was made. Additionally, researchers took note of whether the practice allowed for online appointment scheduling and whether weekend and evening appointment options were offered. An online scheduling system is a web-based application or portal that allows enrollees to conveniently both their appointments through a web-enabled device.

The data collected through the secret shopper survey were compiled and analyzed. Descriptive statistics (mean, range, prevalence) were determined for the following metrics: time until next appointment, practices identified, practices reached, acceptance of appropriate insurance, acceptance of new patients, accuracy of contact information, online booking availability, weekend/evening hour availability, and adherence to AHCCCS network adequacy standards. These metrics were calculated by GSA and insurance type and compared between the Medicaid caller and commercial insurance caller to assess network adequacy.

## 3. Results

Researchers identified, catalogued, and attempted to call a total of 185 unique pediatric practices across the state of Arizona. Accessing the provider directories curated by each MCO, 114 practices were identified and called in Maricopa County, and 18 more across Gila and Pinal counties within the Central GSA; 19 practices were identified in the Northern GSA; and 34 practices in the Southern GSA. Survey results are reported according to each practice's Medicaid GSA designation and displayed in Tables 1 and 2. Across all GSAs, 86% (159) of catalogued pediatric dental practices were reached on behalf of a Medicaid enrolled child and 81% (149) of catalogued pediatric dental practices were reached on behalf of a commercially insured child.

### 3.1. Acceptance of new patients and relevant insurance (see Table 1)

The majority of listed practices were accepting new patients. In the Central GSA, more practices notified the commercial insurer caller (11.5%) that they were not accepting new patients than the Medicaid caller (6.4%). Alternatively, in the South GSA, more practices notified the Medicaid (16.1%) caller that they were not accepting new patients than the commercially insured caller (3.7%).

**Table 1.** Caller experiences of practices by GSA.

	Central Arizona GSA
	Medicaid (Plan A)	Commercial Insurer (Plan B)
Number of Practices Identified	132	132
Practices Reached	83.3%	78.8%
Practices Reached Not Accepting New Patients	6.4%	11.5%
Insurance Accepted	97%	98.8%
Time Until Next Available Appointment, Mean	8.1 days	6.3 days
Time Until Next Available Appointment, Range	0–92 days	0–34 days
	**North Arizona GSA**	
	Medicaid (Plan C)	Commercial Insurer (Plan B and Plan D)
Number of Practices Identified	19	19
Practices Reached	94.7%	94.7%
Practices Reached Not Accepting New Patients	100%	100%
Time Until Next Available Appointment, Mean	5 days	6.6 days
Insurance Accepted	100%	100%
Time Until Next Available Appointment, Range	1–16 days	1–43 days
	**South Arizona GSA**	
	Medicaid (Plan E)	Commercial Insurer (Plan B)
Number of Practices Identified	34	34
Practices Reached	91.2%	79.4%
Practices Reached Not Accepting New Patients	16.1%	3.7%
Insurance Accepted	100%	100%
Time Until Next Available Appointment, Mean	9.3 days	8.3 days
Time Until Next Available Appointment, Range	0–53 days	1–64 days

### 3.2. Next available appointment time (see Table 1)

Researchers calling on behalf of a Medicaid enrolled child experienced longer delays for a practice's next available appointment than those calling on behalf of a child with commercial insurance. However, only 1 practice was found to be in violation of AHCCCS Contractor Operations Manual (ACOM) Policy 417, which requires that networks ensure routine appointments are available within 45 days of request. In this case, the next available appointment was 53 days out.

### 3.3. Factors of accessibility (see Table 2)

Although most practices publish correct contact information, online booking options and weekend appointment availability were offered infrequently and generally limited to practices in large, urban centers (Maricopa and Pima counties). Notably, 4 of the 15 Arizona counties (Apache, Graham, Greenlee, and Santa Cruz) did not have any pediatric dental practices listed in any of the MCO directories. These counties are located in rural parts of the state with high rates of poverty and a high proportion of American Indian and Latino children.

**Table 2.** Factors of accessibility, by AHCCCS GSA.

Accessibility Experience	Prevalence
**Central GSA**
Correct Number Listed	95.4%
Direct Online Booking Available	18.9%
Online Booking Request Available	49.9%
Weekend Availability	25.0%
**North GSA**
Correct Number Listed	100.0%
Direct Online Booking Available	0.0%
Online Booking Request Available	100.0%
Weekend Availability	5.3%
**South GSA**
Correct Number Listed	97.1%
Direct Online Booking Available	2.9%
Online Booking Request Available	41.2%
Weekend Availability	35.3%

## 4. Discussion

An adequate provider network is a critical attribute of health care access. Inadequate networks can prevent patients from being able to see the providers that they know, trust, and depend upon throughout their lives. This is especially crucial for children insured through Medicaid, representing some of the most vulnerable populations and additionally reliant upon both their caregivers and organized health care delivery systems to stay healthy. In an effort to improve our understanding of poor oral health care outcomes for these children, we conducted a direct test of network adequacy among Medicaid dental providers through a “secret shopper” phone survey. This study tested various components of children's access to oral health care, including reliability of provider directory information, appointment availability at the practice level for children covered by both Medicaid and commercial insurance, and compliance with regulatory standards.

The aim of this study was to evaluate just one dimension of access to care. Our findings illuminate some of the marked disparities and barriers in the adequacy of oral health provider networks for children who participate in Medicaid in Arizona. In 4 counties we were unable to identify a single pediatric oral health provider through health plan directories. This finding is particularly alarming and speaks to the need to improve the accessibility of pediatric services in rural regions of the state. We observed minimal differences in appointment wait times between callers with commercial insurance and those insured through Medicaid. This is similar to another secret shopper study that found similar first appointment wait times between Medicaid and privately insured patients seeking primary health care [15].

The unique administration of Arizona's Medicaid program via Managed Care Organizations (MCOs) may play an important role in contextualizing our findings. States entering into contracts with MCOs expect these payers to use their market leverage to negotiate more competitive reimbursement rates with providers in their network in order to meet the needs of the populations they serve. In return, MCOs must be held accountable by states to demonstrate adequate access to care, compliance with federal regulations, and other requirements.

Though AHCCCS requires MCOs to audit and report on the adequacy of their provider networks, enforcement of these requirements and consequences for violations are not transparent. While health plan self-assessment and private accreditation are key components of ensuring network adequacy, it is critical that regulators take a more active role to ensure that network adequacy requirements are evaluated, monitored and enforced. Financial penalties levied against MCOs for falling short of network requirements must also outweigh the costs and financial incentives associated with maintaining viable networks.

A recent Research Brief published by the American Dental Association's Health Policy Institute underscores the complexity of how provider participation is measured and maintained within Medicaid networks. It suggests that in many states, there is a significant portion of Medicaid enrolled providers who do not see any Medicaid patients. A study such as this one, can be useful in further contextualizing data on “access” to determine the actual experiences of Medicaid patients beyond the basic measure of participation, enrollment.

It should be noted that a direct phone call is only one method of scheduling an appointment. The availability and promotion of online scheduling software or other means of scheduling varied between practices. However, such tools can be especially useful to improve accessibility for parents or caregivers who may not be able to place calls during work hours.

Because only providers with a “pediatric” designation merited inclusion in our study population, it is possible that some providers without this designation and catalogued elsewhere in MCO directories could actively be treating patients under 18. However, this would not be immediately distinguishable for parents or dependents attempting to navigate the directories.

Appointment scheduling via direct phone calls can present a significant barrier to utilization of services. Online scheduling platforms could significantly reduce barriers faced by parents trying to schedule appointments during standard business hours or patients who are unable to access a live receptionist. Based on our review, few providers had sophisticated online booking platforms that would reduce barriers to Medicaid patients and their families. AHCCCS and other Medicaid agencies would benefit from requiring, incentivizing, and standardizing use of multilingual online platforms for all practices and providers participating in their network.

## 5. Conclusions

To reduce the number of children with untreated decay, we must improve access to dental care by: educating parents on the importance of early dental visits; sufficiently fund and evaluate systems that support early screening, referral and case management to ensure referrals result in follow up visits; adequately reimbursing health care providers, community health workers, and other trusted community members; and expanding the workforce providing dental care to Arizona's youngest children. Methods and findings here may be replicated or scaled in similar communities nationally to inform efforts to reduce a variety of health disparities, especially among particularly vulnerable populations such as low income rural and rural communities.

Beyond detecting acute occurrences of dental disease, routine oral health screenings can serve as an important opportunity to monitor for signs and risk factors of other physical health conditions that are disproportionately experienced by low-income children. Evaluating and maintaining provider networks takes on an especially important role as we begin to emerge from the COVID-19 pandemic, when more families than ever are relying on Medicaid for health coverage. Facilitating access to routine and recommended oral health screenings for children enrolled in Medicaid is imperative to appropriate stewardship and fulfilling our commitment to provide this vital public health resource.
