# Supplementary material for: Evaluating network adequacy of oral health services for children on Medicaid in Arizona
Source: AIMS Public Health. 2021 Nov 16;9(1):53–61. doi: 10.3934/publichealth.2022005 (PMC8755958; doi:10.3934/publichealth.2022005)
Supplement: Supplementary file 1 [file publichealth-09-01-005-s001.pdf]

Public Health

AIMS Public Health, 9(1): 53–61.
DOI: 10.3934/publichealth.2022005
Received: 13 September 2021
Accepted: 02 November 2021
Published: 16 November 2021

http://www.aimspress.com/journal/aimsph

Research article

Evaluating network adequacy of oral health services for children on Medicaid in Arizona

Swapna Reddy[1,*], Matthew Speer[1], Mary Saxon[1], Madison Ziegler[1], Zaida Dedolph[2] and Siman Qaasim[2]

[1] College of Health Solutions, Arizona State University, Phoenix, AZ, USA
[2] Children's Action Alliance, Phoenix, AZ, USA

* **Correspondence:** Email: Swapna.Reddy@asu.edu; Tel: +2547805284.

Appendix

Survey script

In calls conducted from January through March 2020, 3 Research Assistants (MSa, MZ, LK) followed this standardized set of guidelines outlined in the script below on behalf of a 5 year old patient covered either through an AHCCCS or commercial insurance plan. The script was developed and approved as part of the research protocol filed through the ASU Institutional Review Board.

You are a "secret shopper", presenting on each call as the parent of a 5-year-old child, seeking to schedule a routine dental appointment. Calls should be conducted during normal business hours for each practice, which can be referenced on the provider spreadsheet. Before calling, you will need to

determine what insurers that each office contracts with. This information should be found on their website, and will also be recorded on the provider spreadsheet.

For AHCCCS plans, insurers are categorized according to 3 geographical services areas (GSAs):

- **North** (Mohave, Coconino, Yavapai, Navajo, and Apache)
  - **Indicated Health Plan: Care 1st**
- **Central** (Maricopa, Gila, and Pinal)
  - **Indicated Health Plan: Mercy Care**
- **South** (La Paz, Yuma, Pima, Santa Cruz, Cochise, Graham, Greenlee, and San Carlos Reservation)
  - **Indicated Health Plan: Banner University Family Care**

Please record the following information from each call, if/where applicable:

Date	Practice	Provider	Able to Reach? (Y/N)	Accepting New Patients? (Y/N)	Insurance Accepted? (Y/N)	Date/Time of Soonest Available Appointment

✓ ***Introduction***

Good morning/afternoon! My name is [FIRST/LAST NAME] and I'm calling on behalf of my daughter/son, _____, to schedule a routine teeth cleaning as a new patient with Dr. _____.

✓ ***Age***

_____ is 5 years old.

✓ ***Insurance #1: AHCCCS***

We have [PREDETERMINED AHCCCS PLAN]. Health plan should correspond to the GSA, as shown above.

✓ ***Insurance #2: Commercial/Private***

If asked to provide additional information before scheduling the appointment, such as a detailed medical history for your child or specific insurance identifiers:

_____ doesn't have any allergies, and I believe she/he is up to date on all of her/his vaccinations. I don't have the insurance card with me. Can I bring it with me to the appointment?

NOTE: If you are unable to move forward with scheduling an appointment without detailed insurance information or identifiers, offer to call the office back at a later time to provide this and hang up.

✓ *Appointment*

When is Dr. _____’s next opening? Is there anything available in the morning, evening, or over the weekend?

If/when you are offered an appointment, do not finalize or confirm. End the call with the following:

Perfect. I will just need to give you a call you back later today/tomorrow after I confirm my work schedule. Thank you so much for your time!

After the call has ended, make sure that you have recorded all information in the spreadsheet.

AIMS Press

© 2022 the Author(s), licensee AIMS Press. This is an open access article distributed under the terms of the Creative Commons Attribution License (http://creativecommons.org/licenses/by/4.0)
